# Differences of calcium binding protein immunoreactivities in the young hippocampal CA1 region from the adult following transient ischemic damage

**DOI:** 10.1186/cc12256

**Published:** 2013-03-19

**Authors:** CW Park, HY Lee, JH Cho, MH Won

**Affiliations:** 1 Kangwon National University, Chuncheonsi, South Korea

## Introduction

It has been reported that the young are much more resistant to transient cerebral ischemia than the adult.

## Methods

In the present study, we compared the chronological changes of calcium binding proteins (CBPs) (calbindin 28k (CB-D 28k), calretinin (CR) and parvalbumin (PV)) immunoreactivities and levels in the hippocampal CA1 region of the young gerbil with those in the adult following 5 minutes of transient cerebral ischemia induced by the occlusion of both the common carotid arteries.

## Results

In the present study, we examined that about 90% of CA1 pyramidal cells in the adult gerbil hippocampus died at 4 days post ischemia; however, in the young hippocampus, about 56% of them died at 7 days post ischemia. We compared immunoreactivities and levels of CBPs, such as CB-D 28k, CR and PV. The immunoreactivities and protein levels of all the CBPs in the young sham were higher than those in the adult sham. In the adult, the immunoreactivities and protein levels of all the CBPs were markedly decreased at 4 days post ischemia; however, in the young, they were apparently maintained. At 7 days post ischemia, they were decreased in the young; however, they were much higher than those in the adult.

## Conclusion

In brief, the immunoreactivities and levels of CBPs were not decreased in the ischemic CA1 region of the young 4 days after transient cerebral ischemia. This finding indicates that the longer maintenance of CBPs may contribute to a less and more delayed neuronal death/ damage in the young.

